# Comprehensive TCM treatments combined with chemotherapy for advanced non-small cell lung cancer: A randomized, controlled trial

**DOI:** 10.1097/MD.0000000000025690

**Published:** 2021-05-07

**Authors:** Zhiwei Xiao, Zhiqiang Chen, Rui Han, Liming Lu, Zeyun Li, Jietao Lin, Leihao Hu, Xuewu Huang, Lizhu Lin

**Affiliations:** a Oncology Center, The First Affiliated Hospital of Guangzhou University of Chinese Medicine; b Guangzhou University of Chinese Medicine; c Clinical Research Center, South China Research Center for Acupuncture and Moxibustion, Medical College of Acu-Moxi and Rehabilitation, Guangzhou University of Chinese Medicine, Guangzhou, China.

**Keywords:** chemotherapy, non-small cell lung cancer, randomized controlled trial, traditional Chinese medicine (TCM)

## Abstract

**Objective:**

We conducted this study to evaluate the efficacy and safety of traditional Chinese medicine (TCM) in advanced non-small cell lung cancer (NSCLC) patients who underwent chemotherapy.

**Design:**

This was a prospective, open-label, randomized controlled trial. NSCLC patients at stage IIIA, IIIB, or IV were randomly assigned to either TCM plus chemotherapy or chemotherapy alone. The comprehensive TCM treatment consisted of Kang Ai injection, herbal decoction, and Zhenqifuzheng capsules. The primary endpoint was quality of life (QOL) measured by the Functional Assessment of Cancer Therapy-Lung version 4.0. The secondary endpoints were chemotherapy completion rate, tumor response, and adverse events. All assessments were done at baseline, the third week, and the sixth week.

**Results:**

Thirty-nine participants were randomly assigned to the treatment group and 36 to the control group. The QOL scores were significantly improved in the treatment group compared with those of the control group in social well-being (cycle 1, *P* = .048; cycle 2, *P* = .015), emotional well-being (cycle 1, *P* = .047; cycle 2, *P* = 4.29E-05), and functional well-being (cycle 1, *P* = .030; cycle 2, *P* = .003), while the QOL scores in the above 3 domains declined in the control group (*P* < .05). Both groups had a decline in the physical well-being score (cycle 1, *P* = .042; cycle 2, *P* = .017) and lung cancer symptom score (cycle 1, *P* = .001; cycle 2, *P* = .001) after 2 courses of intervention. The deterioration in physical well-being and lung cancer symptoms was noticeably smaller in the treatment group (*P* < .05). There were significant differences between the 2 groups in social well-being, emotional well-being, functional well-being, lung cancer symptom domain, and the total score (*P* < .05). Patients in the treatment group had a significantly lower incidence of platelet reduction than the control group (*P* = .028) after 2 cycles of treatment. No significant difference in nonhematological adverse events (AEs) was observed.

**Conclusion:**

This study illustrated that comprehensive TCM treatment could promote the QOL of NSCLC patients, alleviate symptoms, and reduce the AEs caused by chemotherapy, verifying the synergistic and attenuating effects of TCM in NSCLC patients undergoing chemotherapy.

**Trial registration:**

Chinese Clinical Trial Registry (www.chictr.org.cn): ChiCTR-TRC-13003637

## 1 Introduction

### 1.1 Description of the condition

Lung cancer is a major factor contributing to cancer-related death worldwide.^[1]^ Non-small cell lung cancer (NSCLC) accounts for 85% to 90% of all cases of lung cancer.^[2]^ Most patients with NSCLC are at an advanced stage when diagnosed, which means that they lose the opportunity for surgery and have to be treated with systemic chemotherapy.^[2,3]^ Although advances have been made in targeted therapy and immunotherapy, most NSCLC patients still possess detrimentally regulated genes or eventually develop acquired resistance. Therefore, platinum-based doublet chemotherapy is the standard treatment for advanced NSCLC.^[4–6]^ However, chemotherapy has met a bottleneck because of its limited efficacy and common toxicity. Up to 75% of patients experience grade 3 or 4 toxicity, sometimes resulting in discontinuation of treatment.^[7]^ High-risk adverse effects such as myelosuppression, fatigue, and anorexia severely affect the quality of life of patients. It is therefore of great interest to improve the efficacy of chemotherapy and reduce side effects.

Traditional Chinese medicine has been widely used in China for cancer patients.^[8,9]^ We have carried out a series of national key scientific and technological research projects on NSCLC,^[10,11]^ which confirmed that traditional Chinese medicine (TCM) could lessen the toxicity of chemotherapy and improve the quality of life (QOL).^[8,12]^ A meta-analysis systematically evaluated the efficacy of Chinese medicine combined with chemotherapy for advanced NSCLC.^[8,12]^ Compared with chemotherapy alone, TCM combined with chemotherapy significantly increased the immediate tumor response and improved the Karnofsky performance score. Combined therapy markedly reduced nausea and vomiting at toxicity grades of III to IV and prevented the decline in hemoglobin and platelets at toxicity grades of I to IV. Cheng also pointed out that the herbal medicine PHY906 can counteract the toxicity of CPT-11 via several mechanisms that act simultaneously.^[13]^

### 1.2 Description of the Intervention

In recent years, comprehensive TCM treatment has become a hot spot in NSCLC research. TCM emphasizes the existence of harmony between the human body, the physical or emotional conditions, and the external environment. Maintaining dynamic homeostasis of the human body is its basic principle.^[14]^ The core pathogenesis of lung cancer in TCM results from a homeostatic imbalance, including disharmony of the spleen and stomach,^[15]^ deficiency of qi and blood,^[14]^ and Yin deficiency of the liver and kidney.^[16,17]^ We followed the corresponding prescriptions to form the TCM treatment decoction in this study, including strengthening the spleen and stomach, nourishing qi and blood, and nourishing the liver and kidney, to restore internal balance.

Some systematic review showed that TCM combined with conventional chemotherapy has advantages in advanced NSCLC patients.^[8]^ However, due to the lack of randomized clinical trials and stratification analysis in the included studies, further rigorous trials are needed. Therefore, we conducted this randomized, controlled study to evaluate the synergistic and attenuating effects of TCM in NSCLC treatment compared with chemotherapy.

## 2 Methods

This was a national, multicentered, prospective, randomized, controlled trial. A total of 75 patients with advanced NSCLC were randomly divided into a treatment group (TCM plus chemotherapy) and a control group (chemotherapy alone).

This study was approved by the Ethical Committee of Guang’anmen Hospital, China Academy of Chinese Medical Sciences (NO.2013EC087-01). This trial was sponsored by the Ministry of Science and Technology of the People's Republic of China and followed the Helsinki Declaration.^[18]^ One of the subcenters, the First Affiliated Hospital of Guangzhou University of Chinese Medicine, has undertaken a large number of research tasks. In this paper, we collated and studied the clinical data of the Guangzhou branch center. All patients provided written, informed consent to participate in this trial.

### 2.1 Inclusion criteria

A pathological or cytological diagnosis of stage IIIa to stage IV NSCLC (using the seventh edition TNM staging system^[19]^); at least 1 measurable lesion according to the Response Evaluation Criteria in Solid Tumors (version 1.1^[20]^); EGFR/ALK-negative cancer or sensitive EGFR/ALK mutations but with apparent intolerance to previous targeted therapies; a plan to receive chemotherapy for at least 2 courses; an Eastern Cooperative Oncology Group performance score of 0 to 2; an estimated survival at least 3 months; age 18 to 75 years old; adequate liver and kidney function; normal hematological function; willingness to participate with written informed consent.

### 2.2 Exclusion criteria

Pregnant or lactating status; only unmeasurable lesions provided, such as pleural effusion, ascites, peritoneal carcinomas, and diffuse bone metastases; any serious concomitant systemic disorder or uncontrollable infection, decompensated heart, lung, or renal failure; chemotherapy intolerable; participation in other clinical trials; allergy to any components of the trial drugs.

### 2.3 Randomization

We used the zone component layer random method in this experiment to stratify the patients according to their gender, TNM stage, pathological type, number of chemotherapy lines, and metastatic sites. A random number table was generated through the CHISS software at a 1:1 ratio in Guang’anmen Hospital, China Academy of Chinese Medical Sciences. Each center competed for enrollment and qualified patients were randomized into the treatment group and the control group according to the order of the random number table. Treatment allocation occurred when the participant met the inclusion criteria and signed the informed consent. The result of randomization was opened to patients and investigators, along with the execution of the study.

### 2.4 Intervention

Patients in the control group were treated with chemotherapy according to National Comprehensive Cancer Network Guidelines in Oncology: Non-Small Cell Lung Cancer (version 1.2012). Patients in the treatment group received comprehensive TCM treatment in addition to chemotherapy. They were administered 3 types of Chinese medicine: herbal injection, herbal decoction, and oral Chinese patent medicine. The specific treatments are summarized in Table 1.

**Table 1:** The specific regimen of treatment.

Group	Regimen	Specific regimen		
Control group	Chemotherapy	TP: Paclitaxel 135 mg/m^2^ on day 1; Cisplatin 75 mg/m^2^ on day 1, or Carboplatin AUC4-6 on day 1		
		DP: Docetaxel 75 mg/m^2^ on day 1; Cisplatin 75 mg/m^2^ on day 1, or Carboplatin AUC4-6 on day 1		
		AP: Pemetrexed 500 mg/m^2^ on day 1; Cisplatin 75 mg/m^2^ on day 1, or Carboplatin AUC4-6 on day 1		
Treatment group	TCM combined with chemotherapy	Herbal injection	Kang Ai injection: 50 mL each day, drip, day 1 to day 10	
		Oral Chinese patent medicine	Strengthening spleen and stomach	XuanFu DaiZhe Tang
			nourishing qi and blood	BaZhen Tang
			nourishing liver and kidney	LiuWei DiHuang Tang
		Herbal decoction	ZhenqiFuzheng capsules: 5 g twice daily for 42 days	

### 2.5 Outcomes and measurements

The primary outcome was QOL obtained by the Functional Assessment of Cancer Therapy-Lung version 4.0 (FACT-L4.0). It has been confirmed that FACT-L4.0 has good reliability, validity, and responsiveness and can be used to measure QOL for Chinese patients with lung cancer.^[21,22]^ There are 36 questions in 5 domains of the FACT-L, which include physical well-being (7 questions), social well-being (7 questions), emotional well-being (6 questions), functional well-being (7 questions), and lung cancer symptoms (9 questions). The questionnaire is in a typical format of a 5-point Likert scale, in which each question ranges from 0 to 4. Positive questions scored forward, and negative questions scored in reverse. The domain score is calculated by summing each question's score. The total score is the sum of each domain score. For each domain and the total score, the higher the score is, the better the QOL of the patients. The QOL data were observed at baseline (week 0) and the third week (week 3) and the sixth week (week 6) of the treatment period.

Secondary outcome measures were the chemotherapy completion rate and tumor response. The tumor response was assessed according to Response Evaluation Criteria in Solid Tumors 1.1 tumor evaluation criteria. The remission rate (RR) and disease control rate (DCR) of the 2 groups were compared at baseline and week 6.

The incidence and severity of all adverse events (AEs) were recorded. Routine urine was collected, and liver and kidney function and electrocardiograms were assessed before and immediately after treatment. Adverse events were defined by grade according to the National Cancer Institute Common Terminology Criteria for Adverse Events version 3.

### 2.6 Statistical analysis

The primary outcome was QOL as measured by FACT-L 4.0. Repeated-measures analysis of variance was used to examine the effect of the interventions (treatment, control) and time (prepost intervention) on QOL. The *t* test was used to analyze the chemotherapy completion rate, tumor response, and assessment of lung cancer symptoms between the treatment group and the placebo group. The baseline characteristics of patients, including sex, age, tumor staging, pathological type, previous treatment history, and TCM syndrome differentiation between the treatment group and control group were compared using the *χ*^2^ test. The number of patients experiencing AEs was compared between the 2 groups using the *χ*^2^ test. We assessed the efficacy of participants who completed 2 courses of medication. Safety outcomes were analyzed in all participants who had received at least 1 cycle of treatment.

An independent third-party CRO company (Beijing Hua Xia Herbal Medicine Technology Co, LTD) conducted the randomization and data management. SPSS19.0 software was used for statistical analysis, and *P* < .05 (2-sided) indicated a significant difference.

## 3 Results

### 3.1 Demographic and baseline clinical characteristics

**Figure 1:**
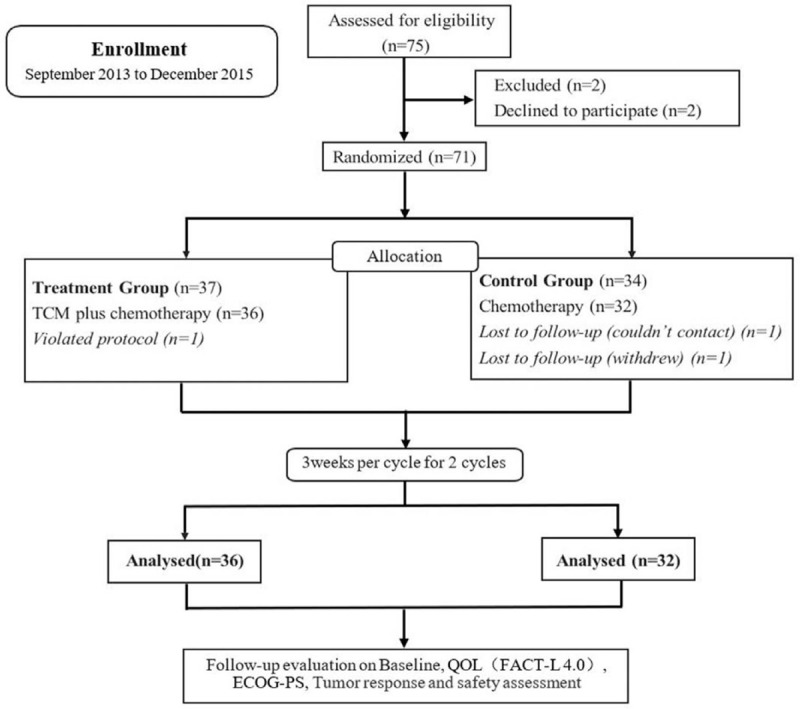
Experimental flow chart.

Between September 2013 and December 2015, a total of 75 eligible patients were enrolled from the cancer center of the First Affiliated Hospital of Guangzhou University of Chinese Medicine. As shown in Figure 1, 2 patients were removed due to meeting the exclusion criteria, and 2 patients declined to participate. Therefore, 71 patients were randomized, including 37 patients in the treatment group and 34 in the control group. During the treatment, 1 patient in the treatment group was excluded due to a violation of the protocol. Two patients in the control group were lost to follow-up. Finally, a total of 68 patients completed the trial according to the protocol and were eligible for full data analysis (Fig. 1).

The baseline characteristics of the treatment group (n = 36) and control group (n = 32) were well balanced in sex, age, tumor stage, pathological type, previous treatment history, and TCM syndrome differentiation. The overall characteristics are listed in Table 2.

**Table 2:** The baseline characteristics of the participants.

Characteristics	Treatment group	Control group	*P* value^∗^
Case, N	36	32	
Mean age (years ± SD)	55.64 ± 9.84	58.50 ± 9.41	.226
Range	36-72	39-78	
Sex			.737
Male	25	21	
Female	11	11	
Metastatic sites			.979
Pulmonary metastases	11	11	
Liver metastases	6	4	
Brain metastases	6	5	
Bone metastases	8	6	
Adrenal metastases	2	1	
Lymphatic metastases	24	23	
Others	8	10	
TNM Stage^†^			.153
IIIA	1	5	
IIIB	7	4	
IV	28	23	
Histological type			.365
Squamous cell carcinoma	12	6	
Adenocarcinoma	21	24	
adenosquamous carcinoma	1	0	
Large cell carcinoma	0	1	
Other	2	1	
Ongoing Chemotherapy			.672
First-line	23	22	
Second-line	13	10	
ECOG PS			.409
0	1	0	
1	25	26	
2	10	6	
TCM Syndromes			.136
Disharmony of spleen and stomach	5	3	
Deficiency of qi and blood	10	7	
Yin deficiency of liver and kidney	21	22	

ECOG = Eastern Cooperative Oncology Group.

∗ *t* test or *χ*^2^ test.

† Based on TNM Classification, the 7th edition.

### 3.2 Comparison of QoL

**Figure 2:**
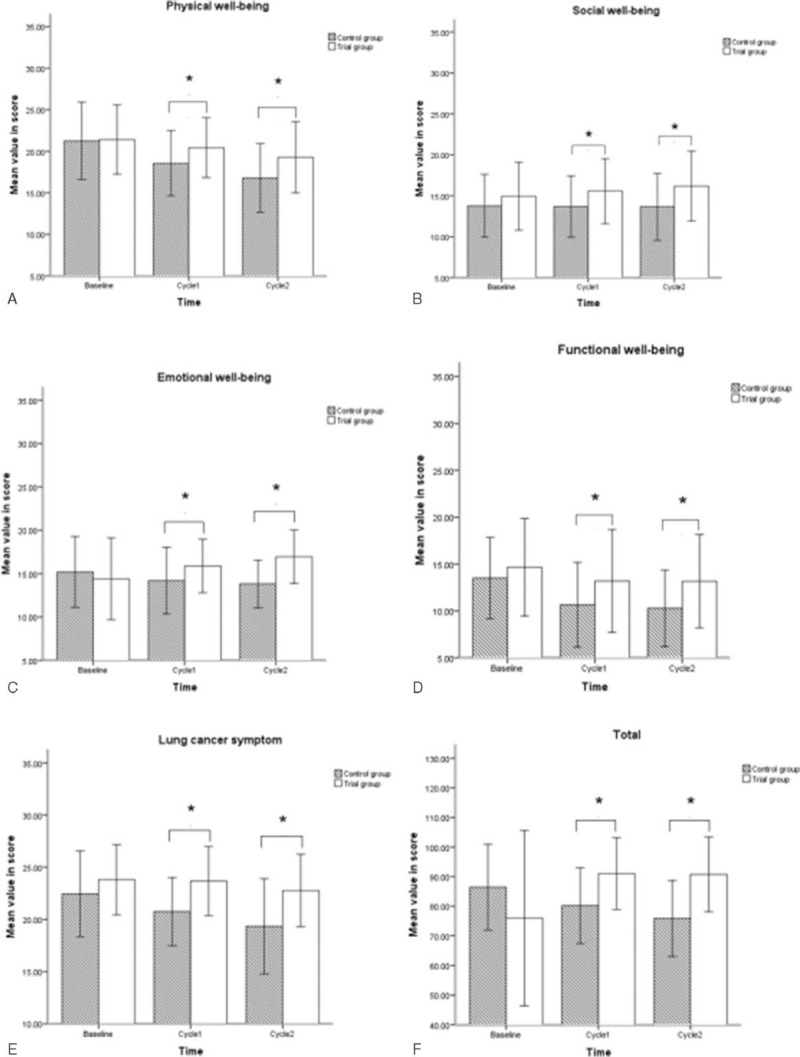
Change in quality of life and comparison of the FACT-L4.0 questionnaire scores in physical well-being, social well-being, emotional well-being, functional well-being, lung cancer symptoms, and total score at baseline, cycle 1, and cycle 2 between the treatment and control groups. FACT-L4.0 = Functional Assessment of Cancer Therapy-Lung version 4.0.

There was no significant difference in any FACT-L subscale score or total score between the 2 groups at baseline (Fig. 2, *P* > .05). Mean scores (mean ± SD) at baseline, at the end of cycle 1, and at the end of cycle 2 in all FACT-L 4.0 subscales in the treatment group and the control group are listed in Table 3. The QOL scores of the treatment group continued to increase over time and were significantly higher than those of the control group in social well-being (cycle 1, *P* = .048; cycle 2, *P* = .015), emotional well-being (cycle 1, *P* = .047; cycle 2, *P* = 4.29E-05), and functional well-being (cycle 1, *P* = .030; cycle 2, *P* = .003). In contrast, a decrease was detected in the control group. There was a decrease over time in the scores on physical well-being and lung cancer symptoms in both groups. Nevertheless, it should be noted that the decline in physical well-being (cycle 1, *P* = .042; cycle 2, *P* = .017) and lung cancer symptoms (cycle 1, *P* = .001; cycle 2, *P* = 9.11 × 10^−6^) associated with the treatment group was significantly smaller than that associated with the control group (*P < *.05).

**Table 3:** Mean scores at baseline, end of cycle 1, and end of cycle 2 in FACT-L 4.0 items between the 2 groups.

FACT-L subscale	Cycle	Treatment group (mean ± SD)	Control group (mean ± SD)	*P* value
Physical well-being	Baseline	21.42 ± 4.17	21.25 ± 4.66	.877
	Cycle 1	20.44 ± 3.58	18.56 ± 3.92	.042
	Cycle 2	19.28 ± 4.29	16.78 ± 4.12	.017
Social well-being	Baseline	14.94 ± 4.14	13.78 ± 3.84	.236
	Cycle 1	15.58 ± 3.97	13.69 ± 3.75	.048
	Cycle 2	16.19 ± 4.27	13.66 ± 4.09	.015
Emotional well-being	Baseline	14.39 ± 4.72	15.19 ± 4.08	.461
	Cycle 1	15.89 ± 3.09	14.19 ± 3.85	.047
	Cycle 2	16.94 ± 3.10	13.81 ± 2.75	4.29E-05
Functional well-being	Baseline	14.67 ± 5.19	13.78 ± 3.97	.437
	Cycle 1	15.42 ± 4.51	13.03 ± 4.31	.030
	Cycle 2	15.58 ± 4.58	12.28 ± 4.08	.003
Lung cancer symptom	Baseline	23.81 ± 3.37	22.44 ± 4.12	.137
	Cycle1	23.67 ± 3.31	20.75 ± 3.27	.001
	Cycle 2	22.78 ± 3.46	19.34 ± 4.57	.001
Total	Baseline	89.22 ± 14.15	86.44 ± 14.58	.427
	Cycle 1	91.00 ± 12.12	80.22 ± 12.82	.001
	Cycle 2	90.78 ± 12.66	75.88 ± 12.86	9.11E-06

FACT-L4.0 = Functional Assessment of Cancer Therapy-Lung version 4.0.

At the third and sixth weeks, the treatment group had higher QOL scores than the control group **(**Fig. 2B–F**)** in social well-being (*P* = .046), emotional well-being (*P* = .04), functional well-being (*P* = .029), lung cancer symptoms (*P* = .001), and the total questionnaire (*P* = .002). However, the score for the physical well-being domain (*P* = .09) barely changed over time and showed no difference between the 2 groups **(**Fig. 2A**)**.

### 3.3 Evaluation of tumor response

The treatment group included no cases of control rate (CR) (0%), 8 cases of partial response (PR) (22.20%), 23 cases of stable disease (SD) (63.90%), and 5 cases of progressive disease (PD) (13.90%). There were 8 cases of RR (22.20%) and 31 cases of DCR (86.10%). In the control group, there were no cases of CR (0%), 8 cases of PR (25.00%), 16 cases of SD (50.00%), 8 cases of PD (25.00%), 8 cases of RR (25.00%), and 24 cases of DCR (75.00%). No significant differences were found in treatment efficacy between the 2 groups (*P* > 0.05) (Table 4).

**Table 4:** Efficacy evaluation of the tumor response.

Group	Treatment group	Control group	χ 2 value	*P* value
N	36	32		
CR	0	0		
PR	8 (22.20)	8 (25.00)		
SD	23 (63.90)	16 (50.00)		
PD	5 (13.90)	8 (25.00)		
RR	8 (22.20)	8 (25.00)	0.073	.788
DCR	31 (86.10)	24 (75.00)	1.353	.245

CR = complete response, DCR = disease control rate, PD = progressive disease, PR = partial response, RR = remission rate, SD = stable disease.

### 3.4 Chemotherapy completion rates

Among the 68 patients, 2 patients in the control group did not complete the second course of chemotherapy due to toxicity, while the rest of the patients completed 2 courses of chemotherapy according to the plan. The completion rates were 94.12% in the control group and 100% in the treatment group. There were no significant differences in the completion rate of chemotherapy between the 2 groups.

### 3.5 Safety

The chemotherapy regimens caused mild (grade 1 or 2) nonhematologic toxic effects in patients: vomiting (cycle 1: 0; cycle 2: 12.50%), fatigue (cycle 1: 21.88%; cycle 2: 28.13%), dry mouth (cycle 1: 6.25%; cycle 2: 15.63%), anorexia (cycle 1: 21.88%; cycle 2: 28.13%), and diarrhoea (cycle 1: 0; cycle 2: 6.25%). These symptoms disappeared 1 week after temporary withdrawal or symptom treatment. There was no significant difference between the 2 groups in nonhematological adverse events (cycle 1: 25.00% vs 31.25%, *P* = .566; cycle 2: 16.67% vs 37.5%, *P* = .052).

Table 5 summarizes the main reported hematological adverse events. After 2 cycles of chemotherapy, we observed changes in values measured by routine blood, liver, and kidney function tests and found that there were no statistically significant differences in leukocytes or hemoglobin between the 2 groups. During the second cycle of chemotherapy, compared with the control, TCM decreased the incidence of platelet reduction (2.78% vs 28.13%, *P* = .003). Moreover, 1 patient experienced liver function lesion in the control group. We did not detect long-term hematological toxicity after symptomatic treatment. No patients quit the trial because of AEs.

**Table 5:** The incidence of hematological toxicity.

		Treatment group (n = 36)			Control group (n = 36)			
		Grade 0	Grade 1 or 2	Grade 3 or 4	Grade 0	Grade 1 or 2	Grade 3 or 4	*P* value
Leukocytes	Cycle 1	33 (91.7%)	2 (5.6%)	1 (2.8%)	27 (84.4%)	5 (15.6%)	0	.352
	Cycle 2	30 (83.3%)	6 (16.7%)	0	24 (75.0%)	5 (15.6%)	3 (9.4%)	.396
Hemoglobin	Cycle 1	18 (50.0%)	18 (50.0%)	0	21 (65.6%)	11 (34.4%)	0	.193
	Cycle 2	17 (47.2%)	18 (50.0%)	1 (2.8%)	15 (46.9%)	17 (53.1%)	0	.977
Platelet	Cycle 1	35 (97.2%)	1 (2.8%)	0	30 (93.8%)	2 (6.3%)	0	.486
	Cycle 2	35 (97.2%)	1 (2.8%)	0	23 (71.9%)	8 (25.0%)	1 (3.1%)	.003

## 4 Discussion

TCM is an experience-based medicine that has been developed in China for thousands of years. TCM is well accepted as a complementary and alternative therapy for NSCLC patients in China. QOL is an important prognostic factor and a significant predictor of the clinical benefit in advanced NSCLC patients.^[23,24]^ The application of the biopsychosocial medical model has made us pay more attention to QOL and not just the time of survival while treating patients with advanced cancer. Multiple clinical studies have confirmed that TCM treatment could promote patient QOL, relieve clinical symptoms, reduce toxicity, and prolong survival time.^[10,12,25]^ However, there is a lack of high-quality trials to prove its efficacy and safety. Thus, we carried out this clinical trial to further verify the synergistic and attenuating effects of TCM in NSCLC patients who are receiving chemotherapy.

Our results showed that QOL improved significantly in patients treated with TCM plus chemotherapy, especially in the social well-being domain, emotional well-being domain, and functional well-being domain. For physical well-being and lung cancer symptoms, the QOL scores decreased after treatment in both groups, which reflected the negative effect of chemotherapy on QOL. Nevertheless, the deterioration was noticeably faster in the control group. This illustrated the efficacy of TCM in maintaining QOL from a different angle. As reported in this study, TCM has special advantages in alleviating symptoms. Patients in the treatment group had a significantly lower incidence of shortness of breath and fatigue. Adverse events were observed in both groups. In this study, the ratio of grade III to IV platelet myelosuppression in the treatment group was significantly lower than that of the control group, but hemoglobin was similar. Moreover, we have not yet seen serious adverse events (SAEs) in either arm during the 2 courses of chemotherapy. All study data suggest that TCM treatment combined with chemotherapy is safe. In general, TCM can effectively improve the quality of life and enable more people to tolerate the toxic side effects of chemotherapy.^[26]^

However, it appeared that TCM did not show outstanding advantages in tumor control, which was consistent with results reported in previous studies. A randomized, controlled, open-label trial showed that the effect of TCM maintenance treatment on time to progression and overall survival (OS) was similar to that of maintenance chemotherapy but improved the quality of life of patients and improved the 1-year survival rate.^[26]^ TCM has obvious long-term therapeutic advantages in the treatment of nonsmall cell lung cancer.

As a unique treatment in China, the anticancer mechanisms of TCM have been widely studied in recent years.^[14,27,28]^ Kang Ai Injection (KAI),^[29–32]^ as one of the main therapeutic drugs in the programme, has been applied as an auxiliary treatment for cancer. A series of anticancer mechanisms have been developed. A meta-analysis found that the combination of KAI injection and systemic chemotherapy may have better efficacy and safety for malignant tumors due to the ability to improve immune function, inhibit tumor cell cloning, arrest the cell cycle, and induce apoptosis in cancer cells.^[30]^ KAI is a Chinese herbal preparation consisting of ginseng, milkvetch root, and kushenin that has been applied as an auxiliary treatment for malignant tumors.^[31]^ A previous study showed that ginseng possesses biological activities, such as antitumor, antihypertensive, antivirus, and immune-modulatory effects.^[33,34]^ Ginsenosides may ameliorate chemoresistance by modulating the NRF2 pathway.^[35]^ Milkvetch roots could increase the activity of T-lymphocyte subsets and NK cells in tumor patients to improve their immunity. CKI is a mixture of natural compounds extracted from Kushen that has been shown to increase immunologic function in advanced NSCLC patients who received chemotherapy.^[35–39]^

There are some limitations to this study. First, the number of patients enrolled may not be sufficient because all data came from a single institution. Thus, the findings might not reflect the whole NSCLC population. Second, we only monitored the patients for 6 weeks, so the follow-up duration of the study may not be long enough. The long-term benefits of TCM for QOL might not be reflected due to the lack of long-term follow-up. In addition, some important endpoints, such as PFS and OS, were not tracked. Our results showed that the completion rate of chemotherapy was not significantly different between the 2 groups. The consistency in chemotherapy tolerance may be related to the short observation period (6 weeks). In future studies, the sample size and intervention time will be expanded, and the patient's PFS and OS will also be observed.

In summary, this study showed that TCM treatment could improve the QOL of NSCLC patients and alleviate their symptoms with good safety. The results of this study provide evidence for TCM application in NSCLC treatment, including the synergistic and attenuating effects of TCM when combined with chemotherapy.

## Author contributions

Zhiwei Xiao drafted the present manuscript, and Zhiqiang Chen, Liming Lu, Zeyun Li, Leihao Hu, Xuewu Huang reviewed and edited the manuscript.

**Data curation:** Zhiqiang Chen, Rui Han, Leihao Hu.

**Formal analysis:** Liming Lu.

**Software:** Zeyun Li, Jietao Lin.

**Statistical design:** Liming Lu, Jietao Lin.

**Supervision:** Xuewu Huang, Lizhu Lin.

**Validation:** Xuewu Huang.

**Writing – original draft:** Zhiwei Xiao

**Writing – review & editing:** Zhiqiang Chen, Jietao Lin, Liming Lu, Zeyun Li, Lizhu Lin.

All authors have read and approved the final manuscript.

## References

[1] Siegel RL Miller KD Jemal A. Cancer statistics, 2019. CA Cancer J Clin 2019;69:07–34. 10.3322/caac.21551 30620402

[2] Duma N Santana-Davila R Molina JR. Non-small cell lung cancer: epidemiology, screening, diagnosis, and treatment. Mayo Clin Proc 2019;94:1623–40. 31378236 10.1016/j.mayocp.2019.01.013

[3] Pujol JL Paz-Ares L de Marinis F. Long-term and low-grade safety results of a phase III study (PARAMOUNT): maintenance pemetrexed plus best supportive care versus placebo plus best supportive care immediately after induction treatment with pemetrexed plus cisplatin for advanced nonsquamous non-small-cell lung cancer. Clin Lung Cancer 2014;15:418–25. 25104617 10.1016/j.cllc.2014.06.007

[4] D’Addario G Pintilie M Leighl NB. Platinum-based versus non-platinum-based chemotherapy in advanced non-small-cell lung cancer: a meta-analysis of the published literature. J Clin Oncol 2005;23:2926–36. 15728229 10.1200/JCO.2005.03.045

[5] Hellmann MD Li BT Chaft JE. Chemotherapy remains an essential element of personalized care for persons with lung cancers. Ann Oncol 2016;27:1829–35. 27456296 10.1093/annonc/mdw271 PMC5035786

[6] Rosenzweig KE Gomez JE. Concurrent chemotherapy and radiation therapy for inoperable locally advanced non-small-cell lung cancer. J Clin Oncol 2017;35:06–10. 10.1200/JCO.2016.69.9678 27870565

[7] Ai D Guan Y Liu XJ. Clinical comparative investigation of efficacy and toxicity of cisplatin plus gemcitabine or plus Abraxane as first-line chemotherapy for stage III/IV non-small-cell lung cancer. Onco Targets Ther 2016;9:5693–8. 27695347 10.2147/OTT.S109683 PMC5033500

[8] Li SG Chen HY Ou-Yang CS. The efficacy of Chinese herbal medicine as an adjunctive therapy for advanced non-small cell lung cancer: a systematic review and meta-analysis. PLoS One 2013;8:e57604. 23469033 10.1371/journal.pone.0057604 PMC3585199

[9] Ye L Jia Y Ji KE. Traditional Chinese medicine in the prevention and treatment of cancer and cancer metastasis. Oncol Lett 2015;10:1240–50. 26622657 10.3892/ol.2015.3459 PMC4533180

[10] Sun L Yim WS Fahey P. Investigation on advanced non-small-cell lung cancer among elderly patients treated with Chinese herbal medicine versus chemotherapy: a pooled analysis of individual data. Evid Based Complement Alternat Med 2019;2019:1898345. 30719055 10.1155/2019/1898345 PMC6334362

[11] Lin LZ Zhou DH Zheng XT. Effect of traditional Chinese medicine in improving quality of life of patients with non-small cell lung cancer in late stage. Chin J Integr Traditional Western Med 2006;26:389–93. 16883901

[12] Chao J Dai Y Verpoorte R. Major achievements of evidence-based traditional Chinese medicine in treating major diseases. Biochem Pharmacol 2017;139:94–104. 28636884 10.1016/j.bcp.2017.06.123

[13] Lam W Bussom S Guan F. The four-herb Chinese medicine PHY906 reduces chemotherapy-induced gastrointestinal toxicity. Sci Transl Med 2010;2:45ra59. 10.1126/scitranslmed.3001270 20720216

[14] Cao J Chen Z Zhu Y. Huangqi-Honghua combination and its main components ameliorate cerebral infarction with Qi deficiency and blood stasis syndrome by antioxidant action in rats. J Ethnopharmacol 2014;155:1053–60. 24960183 10.1016/j.jep.2014.05.061

[15] Huangfu YR Peng W Guo BJ. Effects of acupuncture in treating insomnia due to spleen-stomach disharmony syndrome and its influence on intestinal microbiome: study protocol for a randomized controlled trial. J Integr Med 2019;17:161–6. 30819614 10.1016/j.joim.2019.01.007

[16] Jiang Y Liu LS Shen LP. Traditional Chinese Medicine treatment as adjuvant therapy in completely resected Stage IB-IIIA non-small-cell lung cancer: study protocol for a multicenter, double-blind, randomized, placebo-controlled trial. Clin Lung Cancer 2019;20:e541–7. 31230892 10.1016/j.cllc.2019.05.011

[17] Zhai Y Xu J Feng L. Broad range metabolomics coupled with network analysis for explaining possible mechanisms of Er-Zhi-Wan in treating liver-kidney Yin deficiency syndrome of Traditional Chinese medicine. J Ethnopharmacol 2019;234:57–66. 30690072 10.1016/j.jep.2019.01.019

[18] Vijayananthan A Nawawi O. The importance of Good Clinical Practice guidelines and its role in clinical trials. Biomed Imaging Interv J 2008;4:e5. 21614316 10.2349/biij.4.1.e5 PMC3097692

[19] Edge SB Compton CC. The American Joint Committee on Cancer: the 7th edition of the AJCC cancer staging manual and the future of TNM. Ann Surg Oncol 2010;17:1471–4. 20180029 10.1245/s10434-010-0985-4

[20] Eisenhauer EA Therasse P Bogaerts J. New response evaluation criteria in solid tumours: revised RECIST guideline (version 1.1). Eur J Cancer 2009;45:228–47. 19097774 10.1016/j.ejca.2008.10.026

[21] Cella DF Bonomi AE Lloyd SR. Reliability and validity of the Functional Assessment of Cancer Therapy-Lung (FACT-L) quality of life instrument. Lung Cancer 1995;12:199–220. 7655830 10.1016/0169-5002(95)00450-f

[22] Wan C Zhang C Cai L. Psychometric properties of the Chinese version of the FACT-L for measuring quality of life in patients with lung cancer. Lung Cancer 2007;56:415–21. 17316887 10.1016/j.lungcan.2007.01.004

[23] Montazeri A Milroy R Hole D. Quality of life in lung cancer patients: as an important prognostic factor. Lung Cancer 2001;31:233–40. 11165402 10.1016/s0169-5002(00)00179-3

[24] Xue NZ Fang RM Lin LZ. Application of response evaluation criteria of traditional Chinese medicine for solid tumor in advanced non-small cell lung cancer. Chin J Integr Med 2014;20:910–6. 25428339 10.1007/s11655-014-2022-0

[25] Dong J Su SY Wang MY. Shenqi fuzheng, an injection concocted from Chinese medicinal herbs, combined with platinum-based chemotherapy for advanced non-small cell lung cancer: a systematic review. J Exp Clin Cancer Res 2010;29:137. 20969765 10.1186/1756-9966-29-137 PMC2972256

[26] Jiang Y Liu LS Shen LP. Traditional Chinese Medicine treatment as maintenance therapy in advanced non-small-cell lung cancer: a randomized controlled trial. Complement Ther Med 2016;24:55–62. 26860802 10.1016/j.ctim.2015.12.006

[27] Lam W Jiang Z Guan F. PHY906(KD018), an adjuvant based on a 1800-year-old Chinese medicine, enhanced the anti-tumor activity of Sorafenib by changing the tumor microenvironment. Sci Rep 2015;5:9384. 25819872 10.1038/srep09384 PMC4377583

[28] Saif MW Li J Lamb L. First-in-human phase II trial of the botanical formulation PHY906 with capecitabine as second-line therapy in patients with advanced pancreatic cancer. Cancer Chemother Pharmacol 2014;73:373–80. 24297682 10.1007/s00280-013-2359-7 PMC4123311

[29] Huang S Peng W Mao D. Kangai Injection, a traditional Chinese medicine, improves efficacy and reduces toxicity of chemotherapy in advanced colorectal cancer patients: a systematic review and meta-analysis. Evid Based Complement Alternat Med 2019;2019:8423037. 31379968 10.1155/2019/8423037 PMC6662435

[30] Lu Q Li CL. Therapeutic efficacy and safety of Kang-ai injection combined with platinum-based doublet chemotherapy in advanced NSCLC: a meta-analysis. Life Sci 2018;210:09–19. 10.1016/j.lfs.2018.08.055 30145153

[31] Xue JX Zhu ZY Bian WH. The traditional Chinese medicine Kangai injection as an adjuvant method in combination with chemotherapy for the treatment of breast cancer in Chinese patients: a meta-analysis. Evid Based Complement Alternat Med 2018;2018:6305645. 29849714 10.1155/2018/6305645 PMC5932437

[32] Zhang D Wu J Liu S. Network meta-analysis of Chinese herbal injections combined with the chemotherapy for the treatment of pancreatic cancer. Medicine (Baltimore) 2017;96:e7005. 28538415 10.1097/MD.0000000000007005 PMC5457895

[33] Wang T Fu X Wang Z. Danshen formulae for cancer: a systematic review and meta-analysis of high-quality randomized controlled trials. Evid Based Complement Alternat Med 2019;2019:2310639. 31061667 10.1155/2019/2310639 PMC6466905

[34] Xu J Chu Y Liao B. Panax ginseng genome examination for ginsenoside biosynthesis. Gigascience 2017;6:01–15. 10.1093/gigascience/gix093 PMC5710592 29048480

[35] Chian S Zhao Y Xu M. Ginsenoside Rd reverses cisplatin resistance in non-small-cell lung cancer A549 cells by downregulating the nuclear factor erythroid 2-related factor 2 pathway. Anticancer Drugs 2019;30:838–45. 31415285 10.1097/CAD.0000000000000781

[36] Jin Y Yang Q Liang L. Compound kushen injection suppresses human acute myeloid leukaemia by regulating the Prdxs/ROS/Trx1 signalling pathway. J Exp Clin Cancer Res 2018;37:277. 30454068 10.1186/s13046-018-0948-3 PMC6245615

[37] Xu W Lin H Zhang Y. Compound Kushen Injection suppresses human breast cancer stem-like cells by down-regulating the canonical Wnt/β-catenin pathway. J Exp Clin Cancer Res 2011;30:103. 22032476 10.1186/1756-9966-30-103 PMC3219673

[38] Zhao Z Fan H Higgins T. Fufang Kushen injection inhibits sarcoma growth and tumor-induced hyperalgesia via TRPV1 signaling pathways. Cancer Lett 2014;355:232–41. 25242356 10.1016/j.canlet.2014.08.037 PMC4253542

[39] Zhao Z Liao H Ju Y. Effect of compound Kushen injection on T-cell subgroups and natural killer cells in patients with locally advanced non-small-cell lung cancer treated with concomitant radiochemotherapy. J Tradit Chin Med 2016;36:14–8. 26946613 10.1016/s0254-6272(16)30002-4

